# Geriatric Population During the COVID-19 Pandemic: Problems, Considerations, Exigencies, and Beyond

**DOI:** 10.3389/fpubh.2020.574198

**Published:** 2020-09-22

**Authors:** Kuldeep Dhama, Shailesh Kumar Patel, Rakesh Kumar, Jigyasa Rana, Mohd. Iqbal Yatoo, Akshay Kumar, Ruchi Tiwari, Jaideep Dhama, Senthilkumar Natesan, Rajendra Singh, Harapan Harapan

**Affiliations:** ^1^Division of Pathology, ICAR-Indian Veterinary Research Institute, Bareilly, India; ^2^Department of Veterinary Pathology, Dr. G.C. Negi College of Veterinary and Animal Sciences, GSK Himachal Pradesh Agricultural University, Palampur, India; ^3^Department of Veterinary Anatomy, Faculty of Veterinary and Animal Sciences, Rajiv Gandhi South Campus Banaras Hindu University, Mirzapur, India; ^4^Division of Veterinary Clinical Complex, Faculty of Veterinary Sciences and Animal Husbandry, Sher-E-Kashmir University of Agricultural Sciences and Technology of Kashmir, Srinagar, India; ^5^Department of Cardiothoracic Surgery, Medanta Hospital, Gurgaon, India; ^6^Department of Veterinary Microbiology and Immunology, College of Veterinary Sciences, Uttar Pradesh Pandit Deen Dayal Upadhyaya Pashu Chikitsa Vigyan Vishwavidyalaya Evam Go Anusandhan Sansthan (DUVASU), Mathura, India; ^7^Tara Hospital, New Delhi, India; ^8^Indian Institute of Public Health Gandhinagar, Gandhinagar, India; ^9^Medical Research Unit, School of Medicine, Universitas Syiah Kuala, Banda Aceh, Indonesia; ^10^Tropical Disease Centre, School of Medicine, Universitas Syiah Kuala, Banda Aceh, Indonesia; ^11^Department of Microbiology, School of Medicine, Universitas Syiah Kuala, Banda Aceh, Indonesia

**Keywords:** SARS-CoV-2, COVID-19, geriatrics, older people, vaccines, prevention, disease control

## Abstract

The coronavirus disease 2019 (COVID-19) pandemic wreaked havoc worldwide, with more than 20 million confirmed cases and nearly 0. 75 million deaths as of 10th August 2020. Various factors determine the severity and symptoms of this infection. Older age and underlying diseases are the challenges being faced in controlling and treating COVID-19. In 2019, 703 million of the global population was older than 65 years of age. The estimated mortality due to COVID-19 in people older than 76 years of age is reportedly 18%. Frequent infections in older people, higher disease severity, and increased mortality are major challenges in the implementation of appropriate preventive measures and future strategies to protect against this disease in geriatric population. Poor health status, weak immune function, lowered organ function, increased probability of multiple underlying diseases, and poor attention to personal health can increase the susceptibility to various diseases in the geriatric population. Concerning inadequate immunity, the decrease expression of receptors and exaggerated pathophysiologic responses can be debilitating. However, future studies will reveal the hidden facets in these aspects in this COVID-19 catastrophe. In this article, we reviewed the main concerns of severe acute respiratory syndrome coronavirus 2 (SARS-CoV-2) infection in the geriatric population, including the risk of acquiring severe COVID-19 resulting in mortality, variation in clinical manifestations, and other pandemic-related concerns. We also discussed the need for increasing attention toward the elderly, taking appropriate prevention and control measures, and considering geriatric-related adjustments in vaccine design and development.

## Introduction

Coronavirus disease 2019 (COVID-19) is a highly transmissible disease caused by a novel coronavirus that emerged in Wuhan, China, and was named as severe acute respiratory syndrome coronavirus 2 (SARS-CoV-2) by the International Committee on Taxonomy of Viruses (ICTV) (1). The disease rapidly became globally disseminated and is confirmed to have presently killed nearly 0.75 million people as of 10th August 2020 out of more than 20 million confirmed cases and has triggered a global panic. The pandemic has created a significant impact on the survival and sustenance of human population, with several questions being raised upon the global scientific community as far as the pandemic preparedness is concerned.

The origin of the infection may have been the transmission of SARS-CoV-2 from wild animals available for sale in the Huanan seafood market of Wuhan to humans (2, 3). The pneumonia caused by SARS-CoV-2 was designated as COVID-19 by the World Health Organization (WHO) on 11 February 2020. Bats may be the natural reservoir of the virus, and the search for an intermediate host is underway (4). Pangolins have been suggested as the probable intermediate host. However, there is no conclusive evidence yet (5). Spillover events and zoonotic links have been implicated in the origin of SARS-CoV-2, and the virus infection has been reported from a few animal species (6).

The search for effective therapies and vaccines is going on worldwide (7–10). The large number of fatalities caused by SARS-CoV-2 attests to the severe global impact of the pandemic, irrespective of age, race, sex, and physiological conditions. A report has suggested that everyone will be exposed to the SARS-CoV-2 and that most of the world's population will be infected with COVID-19 (11). Although COVID-19 affects all ages, individuals having comorbidities, such as diabetes, asthma, hypertension, cerebro-cardiovascular abnormalities, cancer, as well as immunocompromised and elderly people, are affected more severely, and exhibit a higher mortality rate (12–14). Age is believed to be a significant determinant of the clinical outcome, severity, disease course, and prognosis of the disease (14, 15). However, many facets need to be discussed. This review highlights SARS-CoV-2 infection in the geriatric population, risk factors, pandemic-related concerns, and the attention required for the elderly. It also considers the development of an effective and safe vaccine for them.

## COVID-19 and Geriatric Population: Global Problem

The elderly, especially those with underlying diseases, are more susceptible for COVID-19 (2, 14, 16, 17). Initial studies of COVID-19 revealed more cases in people 49–55 years of age (2, 16). Subsequent studies involving more people demonstrated that the prevalence of the disease was higher in individuals ≥60 years of age than in younger individuals (14, 18). In developed countries with a very high elderly population, mortality due to COVID-19 was reportedly 83.7% for those >70 years and 16.2% in people younger than 69 years (19). Underlying diseases were noted in 32–51% cases (2, 16). A study also found that SARS-CoV-2 infection is more often associated with detrimental effects in the geriatric population than in younger age groups (20). A retrospective study of 85 patients who had died of SARS-CoV-2 infection in Wuhan reported a median age of the patients was 65.8 years. Among these individuals, underlying non-communicable chronic conditions, such as hypertension, diabetes, and cardiopulmonary diseases, were the most commonly observed comorbidities (21). Most of the patients died due to the multiple organ failure. Clinical manifestations were reportedly more severe and the disease course was more prolonged in the elderly, which required closer monitoring and more medical interventions (14).

## Geriatric Risk Factors for COVID-19

The geriatric population faces special risks for COVID-19 (22). Predisposition and severe outcomes enhance the risks for elderly people (22). Older age and underlying diseases have been noted as the main factors for vulnerability to COVID-19. An age ≥60 years is a major a risk factor (14, 18). Comorbidities are the main underlying etiologies collectively in 32–60% of cases. Specific rates include 16–20% for diabetes, 15–41% for hypertension, and 14–15% for chronic obstructive pulmonary disease and cardiovascular disease (2, 13). Presumably a consequence of advancing age is an inevitable worsening of health related to vital organs. Furthermore, an age-related diminishing physiological functions of multiple organs include the respiratory system and the resulting impaired mucocilary clearance of foreign particles or micro-organisms, is expected (23, 24). Aging alters pulmonary physiology, pathology, and function during lung infections, which affects responsiveness and tolerance in older patients (14, 25). Angiotensin converting enzyme (ACE) 2 expressed on myocytes, renal endothelial cells, and epithelial lung cells acts as a receptor for SARS-CoV-2 (26). Old age has also been associated with weakened physiological functioning of various vital organs and innate/adaptive immune defense. Furthermore, in association with underlying chronic diseases, acquisition of infections is more likely (27). Aging increases the production of interleukin-6 (IL-6) in the brain and the microglia show increased expression of voltage-activated K^+^ channels, potentially enhancing IL-6 production and neuroinflammation with age (28). In innate and adaptive immunity, regulation of membrane potential and calcium influx are determined by the equilibrium potentials of K^+^ (KV1.3, KCa3.1), Na^+^ (TRPM4), and Cl^−^ channels in the plasma membrane. Altered immune function through ion signaling can have profound effects on the increased susceptibility to COVID-19 (29).

Other risk factors include poor nutrition, dementia, dehydration, and various clinical complications, especially in frail and bedridden patients (30). A lack of a timely diagnosis and therapeutic and preventive measures increases the risk of a severe infection.

In addition to compromised organ function and immunity in the elderly, pathophysiological susceptibility also increases their vulnerability, attack rate, and infectivity by SARS-CoV-2 (31). The pneumonia severity index (PSI) score is higher in the elderly than that in the young and middle-aged individuals (15). In one study, the proportion of patients with PSI grade IV and V was significantly greater in the elderly group than that found in the young and middle-aged groups (14). Severe complications due to COVID-19 in older people can include acute respiratory distress syndrome, multiorgan failure, and death, especially in cases with underlying comorbidities (22).

The strict and prolonged lockdown initiated in many locales to prevent the spread of SARS-CoV-2, restricted physical inactivity and produced social isolation-associated stress. These factors may further deteriorate the health of older people, contributing to adverse health outcomes in this population (32). Increased age is a major risk factor for COVID-19 due to various factors including weakened immune system, physical inactivity, and stress. These factors require special attention in addressing the pandemic among the elderly. Social distancing and disconnection can predispose to depression and anxiety in the elderly that may further increase risk of adverse outcomes of COVID-19 (33). In addition, secondary complications due to general care and management also need to be addressed in the elderly. These complications include venous thromboembolism, catheter-related bloodstream infection, pressure ulcers, falls, and delirium (22).

## Clinical Manifestation of COVID-19 in Geriatric Population

The clinical presentation of COVID-19 infection is variable and ranges from a lack of symptoms to symptoms that are mild, severe, and life-threatening. In a study including 72,314 COVID-19 patients, mild, severe, and critical forms of the disease were reported in 81.4, 13.9, and 4.7% of the patients, respectively (20). In addition to fever and cough, dry cough is an important and common clinical manifestation. Coughing is reported in 60–80% of COVID-19 patients (16, 34). Other respiratory symptoms include like dyspnea, sore throat, and rhinorrhea (7, 16, 35, 36). Clinical manifestations have included anorexia, myalgia, asthenia, headache, anosmia, diarrhea, and cardiovascular complications (36, 37). The most common symptom of infection is fever. However, elderly patients frequently have a low intensity fever or no fever, even in severe cases (38). In one study, 77.7% of 18 COVID-19 patients >60 years of age manifested fever. The finding suggests that SARS-CoV-2 infection is not necessarily accompanied by fever (14). Among clinical presentations of COVID-19, presence can differ between elderly and young/middle-aged individuals (14).

SARS-CoV-2 infection reportedly involves elderly men more often than elderly women; however, infection in elderly patients is also reported in Middle East respiratory syndrome (MERS)-CoV (39, 40). Age-specific detailed analysis of COVID-19 symptoms has not been performed. However, the possibility of non-specific and atypical clinical symptoms in elderly patients is highly expected, as is the case in other diseases (41). Moreover, higher frequency of severe disease and mortality is expected along with need for intensive care unit (ICU) hospitalization in elderly patients. The most frequent laboratory hematological finding in critically ill COVID-19 patients is severe lymphocytopenia (<800 cells/μL). This seems to be more pronounced in older patients (14).

Compared to the people <60 years of age, those who are >60 years of age display higher levels of blood urea nitrogen, lactate dehydrogenase activity, and inflammatory indicators (14). The greater involvement of pulmonary lobes in bilateral lesions and more frequent bacterial co-infection have been reported (14). C-reactive protein (CRP) was found to be significantly higher and lymphocyte proportion significantly lower in elderly individuals compared to the CRP and lymphocyte proportion in younger and middle age individuals (15).

## COVID-19 Mortality in Geriatric Population

From what is known, a high death toll among the global geriatric population due to SARS-CoV-2 can be expected. The severe impact of the COVID-19 pandemic is more frequently documented in developed countries with a higher life-expectancy, such as Italy. A 7.2% overall case-fatality rate was reported in Italy, which was significantly greater than the rate of 2.3% in China (42). Age stratification of data revealed a nearly identical case-fatality rate in Italy and China for individuals ≤ 69 years of age. The rate was higher in Italy for those ≥70 years of age, particularly in those ≥80 years of age (42). Moreover, of 1625 fatal cases of COVID-19, 139, 578, and 850 patients were 60–69, 70–79, and ≥80 years of age, respectively (42). Another study in 4021 positive cases indicated a mortality rate of 5.3% in the geriatric population (≥60 years of age) compared with the rate of 1.4% in young and middle-aged individuals (14). In New York, among 5700 hospitalized COVID-19 patients, the in-hospital mortality rate was 15.8, 32.2, 54.3, and 52.3% for adults aged 60–69, 70–79, 80–89, and >90 years, respectively (43).

Older COVID-19 patients with dementia may exhibit mild and atypical symptoms, including diarrhea or drowsiness. However, such old and frail patients have fewer chances to survive the COVID-19 infection. Adequate and appropriate supportive measures, and clinical care may improve their survival rate, even without the use of targeted therapies. Moreover, few COVID-19 patients may die due to worsening of the underlying comorbid health conditions during the infection, rather than by the infection itself (30). In this context, poor nutrition, dementia, dehydration, and other clinical complications are common in frail and bedridden patients, even with mild infective diseases, and well-established risk factors are responsible for worsening health and death, if adequate supportive measures are not provided in time (30).

Immune dysfunction and severity of inflammation are other reasons for increased mortality in COVID-19 patients (2, 16, 44). Antibody-dependent enhancement (ADE) due to cross-reactive antibodies produced in the course of previous infections by other viruses may be a possible cause for this phenomenon (45). Although COVID-19 results in acute respiratory distress syndrome due to acute lung injury in elderly people, which causes many of the deaths, heart attack could be a principle reason for mortality in older people affected with COVID-19, irrespective of the occurrence of pneumonia (46).

Among older COVID-19 patients, higher Sequential Organ Failure Assessment score and elevated d-dimer (>1 μg/mL) were revealed as markers for an increased risk of death. These markers could be used to identify patients with poor prognosis at an early stage (47). Another study involving 179 patients with pre-existing concurrent cardiovascular or cerebrovascular diseases showed an association of high cardiac troponin with a high risk of mortality (21).

## COVID-19 Prevention and Control Measures in Geriatric Population

The current lack of specific vaccines for SAR-CoV-2 or any efficacious vital medications are the main challenges faced in the treatment of COVID-19. Immune-compromised elderly people are especially at risk. An effective vaccine may take more than a year to become widely available. However, considering the rapid pace of vaccine development, there are grounds for optimism concerning the availability of an effective COVID-19 vaccine sooner rather than later (48). In the present scenario, self-quarantine or self-isolation is enforced in most countries to control or mitigate the overwhelming detrimental effects of this pandemic. The recommended measures to prevent the spread of this deadly virus include a regular use of personal protective equipment (PPE), physical distancing, and self-isolation. Social distancing emphasizes reducing the number of cases and preventing community spread. However, this social disconnection has led to an enhanced development of mental deterioration, depression, and suicidal attempts in the geriatric population (49). Self-quarantine during this critical phase of COVID-19 outbreak is specifically oriented toward “social distancing, not social isolation.”

Hand hygiene and respiratory etiquette are also essential recommendations for older people (50). Disinfection of the surroundings in which geriatric people are living should be frequently carried out to prevent contamination of surfaces and reduce chances of infection (50). Healthcare workers, family members, and caregivers of older people should actively implement these basic protocols to prevent the COVID-19 infection among the older population (51).

## Geriatric-Related Considerations and Vaccine Development

Initial results of clinical trials on SARS-CoV-2 spike-based DNA vaccine and inactivated virus vaccine were reported to be safe and induced good neutralizing antibody titers (52, 53). The receptor-binding domain (RBD) of SARS-CoV-2 is reportedly a potential antigen and has been suggested to be a crucial subunit vaccine candidate (54). Moreover, an mRNA-based vaccine (mRNA1273-COVID-19 vaccine) has so-far proven to be safe and is in clinical trial stage (55). A DNA plasmid-based vaccine for COVID-19 designated INO-4800, is being developed by INOVIO Pharmaceuticals, and will be administered by two intradermal injections followed by electroporation (55). Issues with vaccination in older people (who will be the main target of vaccination) include their weaker immune system, which can compromise the recognition and response to novel viruses (56). In addition, amplifying the strength of vaccine may have side-effects in older people and weaker vaccines may require regular boosters/doses. Hence, when vaccines are developed, they will need to be effective for older people (57). Some trials have focused on enrolling older adults for vaccine trials, taking into account the weaker immune system in these individuals. In this context, a Chimpanzee Adenovirus Vector (ChAdOx1)-based vaccine under development for SARS-CoV-2 by the Jenner Institute, Oxford has reached the phase I/II clinical trial stage. Chimpanzee Adenovirus Vector based vaccine is a non-replicating virus and is reported to generate a strong immune response. Thus, it can be safely used in older individuals along with children and individuals with comorbidities (55, 58). Another adenovirus vector-based vaccine (Ad5-nCoV) is among the top contenders for COVID-19 vaccine according to the WHO. However, immunity against Ad5 vector along with safety of the vaccine are major concerns that must be addressed before this vaccine could be used in the geriatric population (59).

SARS-CoV-2 may produce varied pathogenesis, immune responses, and outcomes in older people. The results can include a more severe disease, higher mortality, and prolonged disease (14, 15, 60). In older individuals, a dysfunctional immune system can lead to dysregulated immune response characterized by excessive infiltration of immune cells, cytokine storm, pulmonary edema, pneumonia, widespread inflammation, and multiorgan failure. A healthy immune response usually clears the infection quickly and inactivates the virus with neutralizing antibody with minimal inflammation and tissue damage. However, slower responding, less coordinated, and less efficient immune responses in older individuals can render them more susceptible to emerging infections (60). The inability to switch from innate to adaptive (little to no antibody production) immunity in SARS-CoV infections, especially in older individuals, may need revision (60). In general, most vaccines may not induce effective immune response in older people. However, some vaccines work very well in elderly people (61, 62). For example, the Shingrix vaccine for shingles was found to be 90% effective in people >70 years of age. Immune responses vary greatly among elderly people. Understanding this variability can help in developing new and improved vaccines to protect the most vulnerable elderly people (63). Age-appropriate adjuvants need to be explored (60).

## Special Attention, Welfare, and Motivational Activities During the COVID-19 Pandemic

The incidences of physical violence, discrimination in terms of maltreatment and health care facilities have increased during this global pandemic. However, the pivotal role played by the elderly in retired scientific communities, health workers, and others during this pandemic in terms of sharing their past experiences and providing moral support to those worried about COVID-19 and to their family members cannot be ignored. Geriatrics witnessed World War-2. This experience could help guide policy-making in the aftermath of the current pandemic with the goal of lessening the existing global socioeconomic disparity (64). The WHO must issue different guidelines concerning the special COVID-19 related care of older and geriatric individuals people with disabilities (65). These individuals are vulnerable and they need special attention in the form of social support interventions. The significantly adverse impact of COVID-19 on older people globally, whether they reside in developed or developing countries, can be attributed to a lack of preparedness and the lack of recognition of geriatric health. Fatalities in the geriatric population can be minimized to some extent by providing immediate adequate supportive measures, good health facilities, nursing homes, and care units (66). Geriatrics must be acknowledged and honored for their contributions toward society in their functional life by assisting them to maintain social relationships along with desirable social distancing.

The extensive period of lockdown in nations has made it difficult for some elderly people to obtain food, especially those living alone or those who do not have family members nearby. It is important for citizens, civic bodies, non-governmental organizations, as well as industry leaders to come together and help them in this vulnerable time.

Online platforms need to be explored for betterment of older people so they do not feel isolated and forgotten, to foster a sense of belonging, and to provide social support (67). Older people may not be familiar with online technologies, including smart phones and the internet. To reduce depression and mental stress in the geriatric population, regular behavioral therapy via online motivation and monitoring programs should be implemented. A pilot randomized control trial that assessed the feasibility of reducing loneliness by internet-based cognitive and behavioral interventions reported encouraging results (68). During the current pandemic, elderly people must be motivated to use cell phones, online games, radios, television, to engage in indoor exercise like yoga, and to listen to music (69). Promotion of proper sleep, balanced nutrition, physical activities, and social care in the life style of the geriatric population can reduce the negative effects of SARS-CoV-2 infection. A multidimensional and age-friendly approach with better health care strategies and minimal physical and physiological stressors can be helpful. A number of social welfare programs specifically catering to the elderly should be developed at the local, state, and national levels by various government and non-government organizations. The assistance provided by younger individuals cannot be overstated. This assistance includes running errands, acquiring and delivering groceries, timely provision of medications, and transportation during medical emergencies.

The COVID-19 pandemic has highlighted the need for adequate nursing care for the elderly that is evidence-based and tailored to the needs of this population. A strong public health response and global preparedness to protect the elderly at risk for infectious diseases, including COVID-19, are needed (70). A commentary contributed by 20 international researchers in the field of aging raised the issues of the lack of preparation for crises, such as COVID-19, in long-term care homes and the initial perception of the public that the virus was a problem of elderly people (71). Higher mortality rates among the elderly have devastating consequences in families, as the elderly are a source of generational knowledge and wisdom, and contribute to the workforce critical for the economy and our family (71).

Mental health is also one of the important cornerstones of public health for the elderly. There is a need for regular telephone counseling sessions, contact with family members, provision of relevant and updated information on the pandemic, continued supply of general medications, meeting psychological needs, and instilling a sense of respect and dignity to maintain the health mental status among the elderly (72). The COVID-19 pandemic has revealed the need for a new era of care for older people, including the use of communication technology, more home-based care, and novel approaches to enhance the resilience of the elderly to stress and depression (73). This resilience will build stronger elderly communities with better physical and mental health.

**Figure 1:**
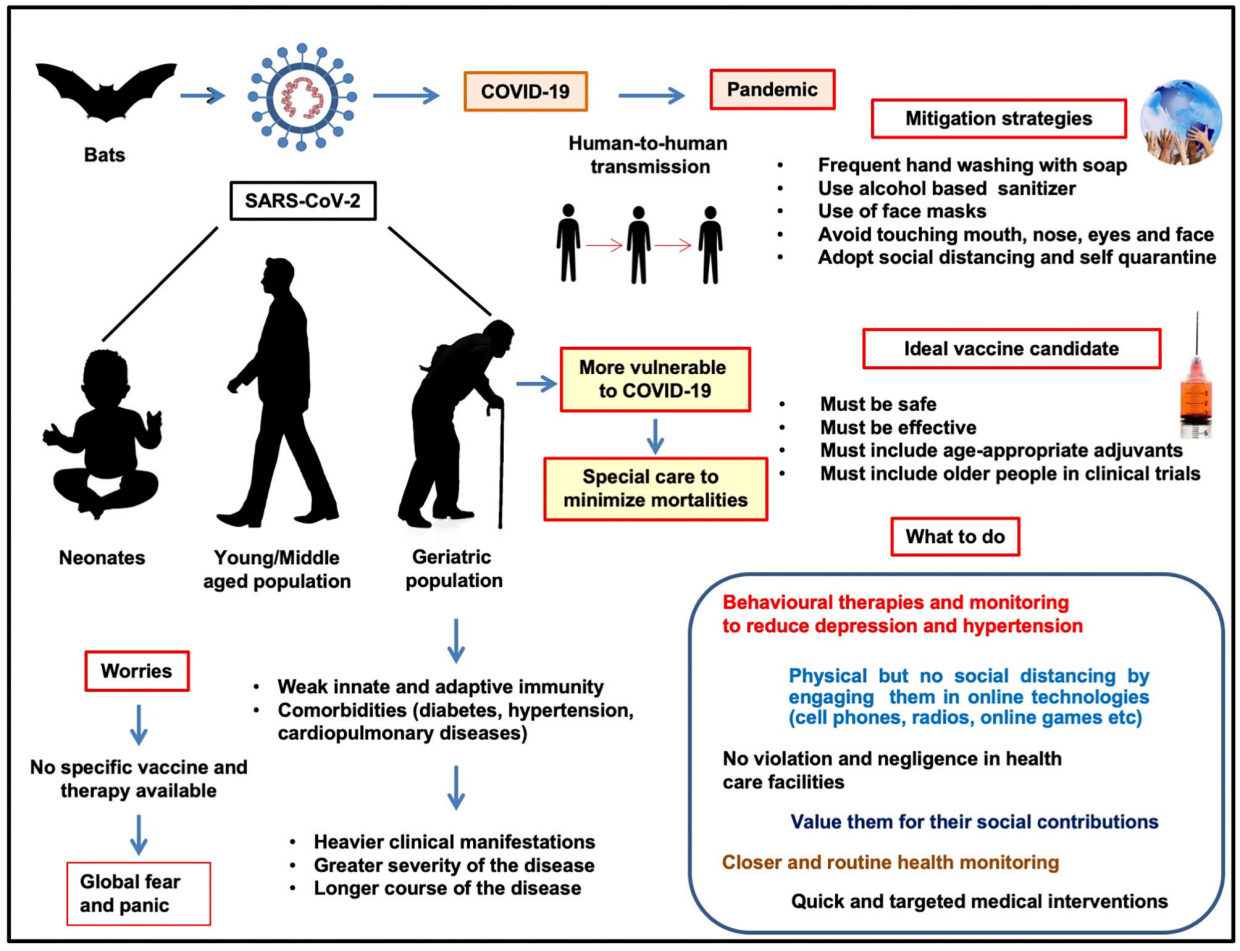
COVID-19 and geriatric population: risk factors and worries, need of special attention during pandemic, development of effective and safer vaccines, and mitigation strategies.

Figure 1 provides an overview of the COVID-19 pandemic in older people, associated risk factors, related worries, need of special attention and care during the pandemic, and the development of effective and safer vaccines and mitigation strategies.

## Conclusion and Future Prospects

The COVID-19 pandemic has spread unimpeded. Millions have been infected with SARS-CoV-2 and over 715,000 hundreds have died. Numerous factors are involved, as reflected in the higher rates of infection in certain classes of society and in different locations. Although individuals of all age groups with diverse physiological conditions are susceptible to infection by the virus, the severity and mortality of COVID-19 is higher in geriatric individuals. Old age, weaker immunity, and underlying diseases are the main predisposing factors for these people. Immunosuppression, decreased organ vitality, and poor healthcare management have increased the suffering of the elderly. Besides increased susceptibility/pathogenicity or infection rate of the virus, dysregulated immune response and hyperinflammation significantly increase the pathophysiology of COVID-19, resulting in higher disease severity and consequently increased mortality in the elderly. Prevention measures need to focus on special requirements of health, nutrition, psychological, and mental well-being of the geriatric population. Physical isolation, rather than social distancing, along with proper hand and respiratory hygiene need to be supported by providing personnel protective equipment, environmental disinfection, and a nutritious diet. Regular behavioral therapy via online motivation and monitoring for older people who are not well-versed with online technologies may reduce depression and mental stress in the geriatric population, and increase their survival. A multidimensional age-friendly approach will certainly minimize physical and physiological stress, and help diminish the toll of the pandemic. Regular monitoring and caring of elderly people will be beneficial in easing COVID-19 related worries, and will facilitate better management of the pandemic. Therapeutics and vaccines must be designed with the elderly in mind to avoid a heavy death toll. Ignorance and insufficient healthcare monitoring and services to the geriatric population may lead to increased mortality. Therefore, health agencies worldwide must pay attention to the geriatric population and issue guidelines specific for this age group.

## Author Contributions

KD: validation, conceptualization, writing—original draft, writing—review and editing, and visualization. SP, RK, JR, MY, AK, RT, JD, SN, and RS: validation and writing—original draft. HH: validation, writing—original draft, and writing—review and editing. All authors: contributed to the article and approved the submitted version.

## Conflict of Interest

The authors declare that the research was conducted in the absence of any commercial or financial relationships that could be construed as a potential conflict of interest.
